# Pre-referral laboratory completeness and diagnostic yield in an early arthritis clinic: a retrospective single-centre analysis

**DOI:** 10.1007/s00296-026-06213-1

**Published:** 2026-07-04

**Authors:** Luisa Schneider, Jörg Christoph Henes, Sebastian Jonas Saur

**Affiliations:** Centre for Interdisciplinary Clinical Immunology, Rheumatology and Autoinflammatory Diseases and Department of Internal Medicine II (Oncology, Haematology, Immunology and Rheumatology), University Hospital Tuebingen, Otfried-Mueller-Strasse 10, Tuebingen, Germany

**Keywords:** Early arthritis clinic, Rheumatoid arthritis, Anti-CCP, Baseline laboratory, Referral pathway, Musculoskeletal ultrasound, DMARD, Methotrexate

## Abstract

To characterise patients presenting to a specialised early arthritis clinic (EAC) at a German university rheumatology centre, and to analyse referral patterns, waiting times, pre-referral laboratory completeness, working diagnoses, serological markers, and independent associates of arthritis confirmation and DMARD initiation. Single-centre retrospective analysis of 290 consecutive patients. At each EAC appointment, a structured assessment comprising medical history, review of available laboratory results, focused physical examination, and arthrosonography was performed. Variables included demographics, serology (RF, anti-CCP), CRP, ESR, imaging, referral source, waiting time, and treatment decisions. Chi-squared, Mann–Whitney U, Kruskal–Wallis tests, and binary logistic regression were performed; p < 0.05 was considered significant. The cohort comprised 212 women (73.1%) and 78 men (26.9%), mean age 49.4 ± 15.9 years. Median waiting time was 23 days (Q1–Q3, 14–40). Although a complete baseline panel (RF, anti-CCP, CRP, ESR) was requested from referring physicians, it was available in only 110 patients (37.9%). Anti-CCP was missing in 34.5%, with missingness significantly associated with referral source (p = 0.013). Inflammatory arthritis was confirmed in 145 patients (50.0%) and DMARD initiation recommended in 130 (44.8%). In multivariable logistic regression (n = 163 with full serology), anti-CCP positivity was independently associated with arthritis confirmation (OR 4.25; 95% CI 1.68–10.74) and DMARD initiation (OR 4.49; 95% CI 1.80–11.22). Orthopaedist referral was independently associated with higher arthritis confirmation (OR 2.97; 95% CI 1.20–7.36). RF was not significantly associated with either outcome. EACs demonstrate high diagnostic yield for inflammatory joint disease. Anti-CCP positivity showed the strongest independent association with both outcomes. Pre-referral laboratory submission was achieved in fewer than 40% of patients despite being a stated referral requirement, with missingness associated with referral source. Targeted educational interventions for referring physicians are warranted.

**Supplementary Information:**

The online version contains supplementary material available at 10.1007/s00296-026-06213-1.

## Introduction

Rheumatoid arthritis (RA) and related inflammatory arthritides cause substantial joint destruction and functional disability when diagnosis and treatment are delayed [1]. The therapeutic "window of opportunity" – the period during which early DMARD initiation yields superior disease modification – is supported by systematic review evidence showing that earlier commencement is associated with reduced radiographic progression and higher rates of sustained remission [2, 3]. A landmark single-centre study confirmed greater improvement in disease activity and slower radiographic progression in patients commencing DMARDs at three months versus twelve months from symptom onset [4].

Specialised early arthritis clinics (EACs) have been established to ensure rapid expert assessment and timely therapy for patients with new-onset joint symptoms [5]. Structured referral pathways with triage criteria improve diagnostic yield: one German single-centre analysis demonstrated nearly twice the rate of confirmed inflammatory disease in EAC-triaged patients compared with standard elective referrals (49.2% vs 26.2%; p < 0.001) [6]. Despite this, delays from symptom onset to specialist assessment remain a recognised international challenge [7].

The diagnostic evaluation of early inflammatory arthritis relies on the 2010 ACR/EULAR classification criteria integrating joint involvement, serology (RF and anti-CCP), acute-phase reactants (CRP and ESR), and symptom duration [8], supplemented by arthrosonography. Musculoskeletal ultrasound (MSUS) detects subclinical synovitis, tenosynovitis, and enthesitis with a high negative predictive value (~ 89%) for progression to inflammatory arthritis [9], and MRI identifies bone marrow oedema as an independent predictor of erosive progression [10].

Previous studies have reported EAC diagnostic yields and patient characteristics in various European centres [4–6], but systematic data on pre-referral laboratory completeness as a quality indicator of the referral process – and its association with diagnostic outcomes – are lacking from German university EAC settings. We therefore conducted a retrospective analysis of 290 patients presenting to the EAC of University Hospital Tübingen, examining patient characteristics, pre-referral laboratory completeness and its determinants, serological and clinical associates of arthritis confirmation and DMARD initiation, and the working diagnoses generated by integrated clinical and sonographic assessment.

## Methods

### Study design and setting

This is a single-centre, retrospective, non-interventional cohort analysis, reported in accordance with the STROBE statement for observational studies [15]. Patients were consecutively registered at the Früharthritis-Sprechstunde (FAS), Rheumatologische Ambulanz, Medizinische Klinik II, University Hospital Tübingen, Germany. The study was approved by the Ethics Committee of the Medical Faculty, University of Tübingen (Ethikkommission der Medizinischen Fakultät der Universität Tübingen; Project No. 122/2025BO2; approved 11 March 2025). Given the fully anonymised, retrospective nature of the analysis, the requirement for individual written informed consent was waived by the Ethics Committee, consistent with applicable German research ethics regulations. All procedures comply with the 1964 Declaration of Helsinki and its later amendments.

### Referral procedure and pre-referral laboratory requirements

The EAC operates as an open-access specialist service for patients with new or undifferentiated joint complaints. At registration, referring physicians – general practitioners (GPs), orthopaedists, and other specialists – were requested as part of the referral protocol to submit a baseline laboratory panel comprising rheumatoid factor (RF), anti-cyclic citrullinated peptide antibodies (anti-CCP), C-reactive protein (CRP), and erythrocyte sedimentation rate (ESR) prior to the appointment, consistent with EULAR early arthritis management recommendations [11].

### Diagnostic procedure at the EAC

Each EAC appointment followed a standardised structured protocol. After review of the referral letter and available pre-referral laboratory results, a focused medical history was obtained covering symptom onset, duration, joint distribution, morning stiffness, and relevant comorbidities including psoriasis and family history. This was followed by a targeted physical examination with systematic joint assessment. Arthrosonography (musculoskeletal ultrasound, MSUS) was performed using a standardised protocol evaluating B-mode greyscale synovitis (grades 0–3, EULAR OMERACT), power Doppler signal (grades 0–3), tenosynovitis, enthesitis, and joint effusion in a joint set determined by the clinical presentation. Examinations were performed by the attending consultant rheumatologist [9, 13, 14].

Based on the integrated assessment of medical history, available serology, focused physical examination, and MSUS findings, a working diagnosis was established at each appointment by the attending rheumatologist. Conventional radiography was performed to assess for erosive changes and structural pathology; MRI was employed in seronegative patients with clinical and sonographic suspicion of inflammatory arthritis to detect bone marrow oedema. Where a DMARD indication was identified, first-line treatment was initiated according to current EULAR recommendations, most commonly methotrexate with short-term glucocorticoids.

### Outcome definitions

"Inflammatory arthritis confirmed" was defined as the attending rheumatologist recording a diagnosis of inflammatory arthritis (including RA, spondyloarthritis, psoriatic arthritis, polymyalgia rheumatica, crystal arthropathy, connective tissue disease, or undifferentiated inflammatory arthritis) based on the integrated EAC assessment. Diagnoses of arthralgia, osteoarthritis, fibromyalgia, or other non-inflammatory conditions were classified as not confirmed. ACR/EULAR 2010 classification criteria were considered where applicable but were not strictly required for working diagnoses [8, 16].

"DMARD indication" was defined as the attending physician documenting a recommendation to initiate or escalate conventional synthetic or biological DMARD therapy at the EAC appointment. Both outcomes reflect clinician judgment at the time of the first specialist assessment and are therefore subject to inter-physician variability and evolving diagnostic certainty over time; this is acknowledged explicitly as a study limitation.

### Data collection

Extracted variables: sex; date of birth (for age calculation); date of registration and appointment (for waiting time); completeness of the pre-referral laboratory panel (all four markers present, yes/no); individual serological values (RF: positive/negative/not available; anti-CCP: positive/negative/not available); CRP (mg/dL) and ESR (mm/h); working diagnosis; imaging performed (none, radiography, MRI, or both); joint aspiration; referral source (GP, orthopaedist, other); arthritis confirmed (yes/no); DMARD initiation recommended (yes/no); appointment duration (minutes); smoking status; and symptom duration (≤ or > 6 months). Quantitative sonographic scores were not systematically recorded in the retrospective database and therefore could not be included as predictors.

### Statistical analysis

Continuous variables: mean ± SD and median with first and third quartiles (Q1–Q3); where reported, the interquartile range (IQR) is the single value Q3 − Q1. Categorical variables: n (%). Comparisons: Mann–Whitney U test (two groups), Kruskal–Wallis test (≥ 3 groups), chi-squared test (categorical associations). Missingness of serological markers was analysed by chi-squared test (by referral source and arthritis status) and Mann–Whitney U test (by waiting time) to assess whether data were missing completely at random (MCAR).

Multivariable logistic regression was performed for both primary outcomes. Model A (n = 287, no serology) included sex, age, waiting time, referral source (orthopaedist vs GP [reference]; other vs GP), and symptom duration < 6 months. Model B (n = 163, with serology) additionally included anti-CCP (positive/negative) and RF (positive/negative), restricted to patients with complete serological data. Results are reported as odds ratios (OR) with 95% confidence intervals (CI). A two-sided p < 0.05 was considered statistically significant. Analyses were performed in Python 3.12 (SciPy 1.11, statsmodels 0.14) by the authors; no external professional statistician was involved, and the statistical analyses were carried out and independently checked by the authors themselves.

## Results

### Patient characteristics

**Fig. 1:**
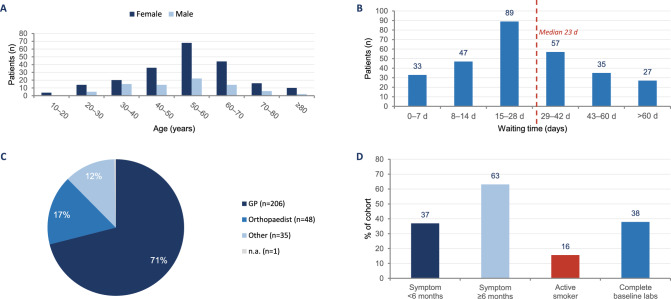
**A** Age distribution by sex: 50–60 years most frequent; males significantly older. **B** Waiting time: median 23 days (IQR 14–40); dashed line = median. **C** Referral source: GPs 71.0%. **D** Complete pre-referral laboratory panel available in only 37.9%. *IQR* interquartile range. Complete baseline labs = RF + anti-CCP + CRP + ESR all present at appointment. Age male vs female: Mann–Whitney U, p = 0.0001

A total of 290 patients were included (Table 1; Fig. 1). The cohort comprised 212 women (73.1%) and 78 men (26.9%). Mean age was 49.4 ± 15.9 years (median 51.3; Q1–Q3, 37.1–60.5). Male patients were significantly older than female patients (55.4 ± 15.4 vs 47.3 ± 15.5 years; p = 0.0001, Mann–Whitney U), consistent with the known higher prevalence of late-onset inflammatory arthritis in men [12]. Smoking was reported in 45 patients (15.6%). Symptom duration of less than 6 months at presentation was documented in 107 patients (36.9%).

**Table 1:** Baseline characteristics of all 290 consecutive patients

Variable	Value	Missing (n)
Total patients	290 (100%)	—
Female, n (%)	212 (73.1%)	0
Male, n (%)	78 (26.9%)	0
Age, mean ± SD (years)	49.4 ± 15.9	3
Age, median (Q1–Q3, years)	51.3 (37.1–60.5)	
Age, female – mean ± SD	47.3 ± 15.5	
Age, male – mean ± SD	55.4 ± 15.4	
Active smoker, n (%)	45 (15.6%)	1
Symptom duration < 6 months, n (%)	107 (36.9%)	0
Inflammatory arthritis confirmed, n (%)	145 (50.0%)	0
DMARD initiation recommended, n (%)	130 (44.8%)	0
Appointment duration, mean ± SD (min)	22.0 ± 6.6	0

The cohort is predominantly female (73.1%). Male patients were significantly older (55.4 vs 47.3 years; p = 0.0001). A DMARD recommendation was documented in 44.8%

*Q1–Q3* first to third quartile, *SD* standard deviation, *DMARD* disease-modifying antirheumatic drug

Age male vs female: Mann–Whitney U, p = 0.0001

### Referral patterns and waiting times

Most patients were referred by GPs (n = 206; 71.0%), followed by orthopaedists (n = 48; 16.6%) and other specialists (n = 35; 12.1%). Median waiting time from registration to appointment was 23 days (Q1–Q3, 14–40; mean 28.6 ± 19.8; range 0–94 days). No significant differences in waiting time were observed between referral sources (Kruskal–Wallis H = 0.92; p = 0.633). Patients with confirmed inflammatory arthritis had a shorter but not significantly different waiting time compared with those without (median 21 vs 27 days; p = 0.198), possibly reflecting more urgent referral for patients with overt synovitis [4, 5].

### Pre-referral laboratory completeness

**Fig. 2:**
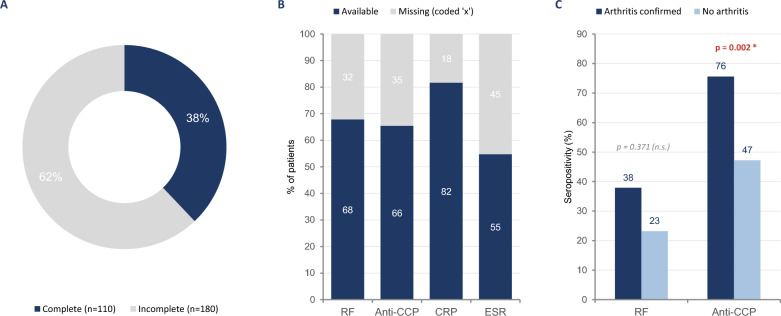
**A** Complete pre-referral panel in only 37.9%. **B** CRP most consistently submitted (81.7%); ESR most frequently missing (45.2%). **C** Anti-CCP positivity significantly higher with confirmed arthritis (76% vs 47%; p = 0.002); RF: p = 0.371 (n.s.). Missing = coded 'x' at time of appointment. Chi-squared test; * p < 0.05. CRP availability corrected: 81.7% present (53/290 missing, not 0)

Despite the pre-referral laboratory panel being requested from all referring physicians [11], the complete four-marker panel (RF, anti-CCP, CRP, ESR) was available in only 110 of 290 patients (37.9%) at the time of the EAC appointment. RF was unavailable in 93 patients (32.1%), anti-CCP in 100 (34.5%), CRP in 53 (18.3%), and ESR in 131 (45.2%; Table 3; Fig. 2A–B).

Formal missingness analysis revealed that anti-CCP missingness was significantly associated with referral source: anti-CCP was absent in 31.6% of GP-referred patients, 31.3% of orthopaedist-referred patients, but 57.1% of patients referred from other specialists (chi-squared p = 0.013). Waiting time did not predict anti-CCP missingness (median 25 [missing] vs 23 days [present]; Mann–Whitney U p = 0.884), nor did arthritis status (missing in 40.0% of non-arthritis vs 29.7% of arthritis patients; p = 0.077). A similar pattern was observed for RF (referral source p = 0.141; waiting time p = 0.935). These data suggest the missing data mechanism is not completely at random (not MCAR) and is primarily driven by referral source rather than disease severity (Table 2).

**Table 2:** Pre-referral availability of the four requested laboratory markers (n = 290)

Marker	Available, n (%)	Missing ('x'), n (%)
Rheumatoid factor (RF)	197 (67.9%)	93 (32.1%)
Anti-cyclic citrullinated peptide antibodies (anti-CCP)	190 (65.5%)	100 (34.5%)
C-reactive protein (CRP)	237 (81.7%)	53 (18.3%)
Erythrocyte sedimentation rate (ESR)	159 (54.8%)	131 (45.2%)
Complete panel (all four markers present)	110 (37.9%)	180 (62.1%)

"Available" = result present at the appointment; "Missing" = recorded as not available (coded 'x'). The complete panel comprises all four markers simultaneously. Anti-CCP missingness was associated with referral source (p = 0.013) but not waiting time (p = 0.884) or arthritis status (p = 0.077)

### Univariate associations of serological markers with clinical outcomes

Among patients with available results, RF was positive in 52 of 197 tested (26.4%) and anti-CCP in 45 of 189 tested (23.8%). In univariate analyses (Table 3), anti-CCP positivity was significantly associated with confirmed inflammatory arthritis (75.6% vs 47.2%; χ^2^ = 9.97; p = 0.002) and DMARD initiation (73.3% vs 40.3%; χ^2^ = 13.71; p < 0.001; Fig. 2C). RF positivity showed no significant univariate association with either outcome (arthritis p = 0.371; DMARD p = 0.207) [8, 11].

**Table 3:** Univariate associations of serological status with clinical outcomes (chi-squared test)

Marker / Status	n tested	Arthritis rate	p (arthritis)	DMARD rate	p (DMARD)
Anti-CCP positive	45	75.6%	0.002	73.3%	< 0.001
Anti-CCP negative	144	47.2%	ref	40.3%	ref
RF positive	52	55.8%	0.371	53.8%	0.207
RF negative	144	47.2%	ref	42.4%	ref

Rates calculated only among patients with an available result (anti-CCP, n = 189; RF, n = 196). Multivariable-adjusted estimates are shown in Table 4

*anti-CCP* anti-cyclic citrullinated peptide antibodies, *DMARD* disease-modifying antirheumatic drug, *ref*. reference category, *RF* rheumatoid factor

### Working diagnoses

Following integrated assessment, a working diagnosis was established in all 290 patients (See Supplemental Material). The most frequent were arthralgia/unspecified arthralgia (n = 84; 29.0%), RA (n = 65; 22.4%), osteoarthritis (n = 47; 16.2%), spondyloarthritis (n = 28; 9.7%), psoriatic arthritis (n = 26; 9.0%), polymyalgia rheumatica (PMR; n = 14; 4.8%), and crystal arthropathy/gout (n = 8; 2.8%). Grouped by category, inflammatory working diagnoses accounted for 47.6% of the cohort (RA 22.4%, spondyloarthritis 9.7%, psoriatic arthritis 9.0%, PMR 4.8%, connective tissue disease 1.0%, undifferentiated 0.7%), degenerative disease (osteoarthritis) for 16.2%, non-inflammatory conditions (arthralgia and fibromyalgia) for 31.0%, metabolic disease (crystal arthropathy/gout) for 2.8%, and other diagnoses for 2.4%. The marginally higher proportion with a confirmed inflammatory arthritis outcome (50.0%, n = 145; Table 1) reflects that this binary clinical determination additionally captured a small number of patients whose working diagnosis was initially non-specific but who were judged to have inflammatory disease. This broad diagnostic spectrum reflects the heterogeneity of EAC referrals and the importance of integrated clinical and sonographic assessment for efficient differential diagnosis [9].

### Multivariable analysis

**Fig. 3:**
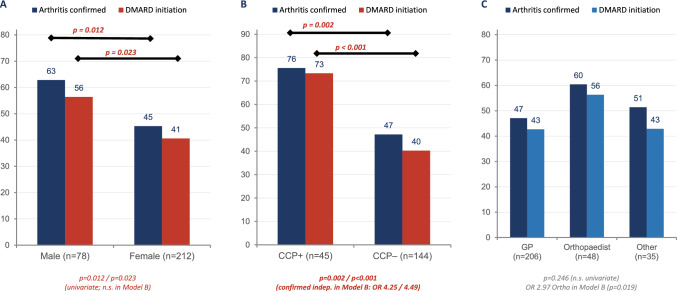
Univariate associations (chi-squared). For multivariable-adjusted ORs see Table 4. **A** Male sex: higher arthritis (63% vs 45%; p = 0.012) and DMARD rates (56% vs 41%; p = 0.023) univariately; not significant in multivariable Model B. **B** Anti-CCP positivity: highest arthritis (76% vs 47%; p = 0.002) and DMARD rates (73% vs 40%; p < 0.001); confirmed as independent factor in Model B (OR 4.25 and 4.49). **C** Referral: orthopaedist numerically highest (60%); p = 0.246 univariate; OR 2.97 in Model B (p = 0.019). Univariate chi-squared test. For multivariable-adjusted OR see Table 4. *DMARD *disease-modifying antirheumatic drug; *n.s.* not significant. Serology: tested patients only

Binary logistic regression was performed for both primary outcomes. Model A (n = 287, without serology) and Model B (n = 163, with serological data) are presented in Table 4 and Fig. 3.

**Table 4:** Both models are reported: Model A addresses clinical predictors in the full cohort; Model B adds serology but is restricted to patients with available results (n = 163)

Variable	n	OR	95% CI	Model
*Arthritis confirmed*				
Male sex	287	1.79	1.02–3.16	A (clinical predictors)
Symptom duration < 6 months	287	1.80	1.08–2.98	A (clinical predictors)
Age (per year)	287	1.00	0.99–1.02	A (clinical predictors)
Orthopaedist referral	287	1.58	0.81–3.06	A (clinical predictors)
Anti-CCP positive	163	4.25	1.68–10.74	B (+ serology)
Orthopaedist referral	163	2.97	1.20–7.36	B (+ serology)
Male sex	163	1.80	0.82–3.94	B (+ serology)
RF positive	163	0.96	0.40–2.29	B (+ serology)
*DMARD initiation recommended*				
Male sex	287	1.70	0.97–2.98	A (clinical predictors)
Anti-CCP positive	163	4.49	1.80–11.22	B (+ serology)
Orthopaedist referral	163	3.25	1.32–8.04	B (+ serology)
Male sex	163	1.92	0.87–4.24	B (+ serology)
RF positive	163	1.11	0.46–2.70	B (+ serology)

In Model B, anti-CCP positivity is the strongest independent associate of both outcomes. Male sex, significant in Model A (arthritis: OR 1.79; 95% CI 1.02–3.16), did not reach significance in Model B, indicating partial mediation by serological status

Model A: n = 287 (sex, age, waiting time, referral source, symptom duration; no serology). Model B: n = 163 (adds anti-CCP + RF; complete-case)

Associations with a 95% CI excluding 1.0 are statistically significant

*OR *odds ratio, *CI* confidence interval

In Model A, male sex was independently associated with arthritis confirmation (OR 1.79; 95% CI 1.02–3.16) and symptom duration < 6 months also showed an independent association (OR 1.80; 95% CI 1.08–2.98). In Model B (with serology), anti-CCP positivity was the strongest factor independently associated with arthritis confirmation (OR 4.25; 95% CI 1.68–10.74) and DMARD initiation (OR 4.49; 95% CI 1.80–11.22). In Model B, male sex no longer reached significance for either outcome (95% CI 0.82–3.94 for arthritis; 95% CI 0.87–4.24 for DMARD, both crossing unity), indicating that the univariate sex association is partly explained by differences in serological status and case-mix. Orthopaedist referral was independently associated with higher arthritis confirmation in Model B (OR 2.97; 95% CI 1.20–7.36). RF was not significantly associated with either outcome in either model.

### Treatment

A DMARD recommendation was documented in 130 patients (44.8%). In accordance with EULAR 2022 recommendations [11], methotrexate combined with a standard glucocorticoid tapering regimen was the most frequently recommended first-line therapy. Joint aspiration was performed in 15 patients (5.2%): diagnostic in 4, therapeutic in 3, and combined in 8 cases. One patient required inpatient admission (0.3%). Imaging beyond arthrosonography was performed in 120 patients (41.4%): MRI alone n = 59 (20.3%), radiography alone n = 43 (14.8%), both n = 18 (6.2%).

## Discussion

This retrospective analysis of 290 consecutive patients presenting to a university-based EAC demonstrated a 50.0% inflammatory arthritis confirmation rate through integrated clinical, serological, and sonographic assessment. This is consistent with published European EAC data reporting diagnostic yields of 30–55%, and higher than rates reported for standard elective rheumatology referrals [5, 6].

Added value over prior literature: to our knowledge, this is the first systematic German university EAC analysis to report pre-referral laboratory completeness as a formal quality indicator of the referral process. Prior publications, including the largest German single-centre dataset (Krause et al. [6]), have not systematically reported laboratory submission rates or formally analysed missingness by referral source. The finding that fewer than 38% of patients present with a complete panel, and that missingness differs by referral source, has direct practical implications for EAC workflow design that have not been previously quantified.

The diagnostic protocol at our EAC integrates medical history, pre-referral laboratory review, focused physical examination, and arthrosonography [9, 13, 14]. MSUS plays a pivotal role: detecting subclinical synovitis, tenosynovitis, and enthesitis with high sensitivity and a negative predictive value approaching 89% for progression to inflammatory arthritis in clinically suspect presentations [9]. The broad diagnostic spectrum – including inflammatory working diagnoses (47.6%), degenerative disease (16.2%), and non-inflammatory conditions such as arthralgia and fibromyalgia (31.0%) – reflects the heterogeneity of EAC referrals and underscores the need for integrated multimodal assessment.

A central finding is that pre-referral laboratory completion was achieved in only 37.9% of patients despite being requested as part of the referral protocol, and missingness was associated with referral source (p = 0.013) but not with waiting time or disease severity. Patients referred by "other specialists" had the highest anti-CCP missingness rate (57.1%). The mechanism of missingness is not completely at random (not MCAR), and the serological complete-case analyses (n = 163) may therefore underestimate or overestimate effect sizes if patients with missing serology systematically differ from those tested. These findings point to a significant gap between clinical protocol requirements and actual practice, highlighting the need for structured educational interventions for GPs and orthopaedists [7].

In multivariable logistic regression, anti-CCP positivity showed the strongest independent association with both arthritis confirmation (OR 4.25; 95% CI 1.68–10.74) and DMARD initiation (OR 4.49; 95% CI 1.80–11.22). This is consistent with the established high specificity of anti-CCP for RA and its role as a predictor of an erosive disease course [8, 11]. RF was not significantly associated with either outcome in any model, likely reflecting its lower disease specificity. Importantly, the anti-CCP association reported here was derived from a pre-selected EAC population with clinical suspicion of inflammatory arthritis, and these associations cannot be extrapolated to primary care or general outpatient settings. Quantitative MSUS scores and imaging findings contributed clinically to outcome determination but were not captured as predictors in this retrospective dataset, representing a significant analytical limitation.

Male sex showed an independent association with arthritis confirmation in Model A (OR 1.79; 95% CI 1.02–3.16), but this was not significant when serological status was added in Model B (95% CI 0.82–3.94, crossing unity). This indicates that the univariate sex effect is at least partly explained by differences in anti-CCP positivity rates and case-mix, consistent with the known contribution of late-onset RA (LORA) and PMR – conditions with more equal sex distributions – in older male patients [12]. Orthopaedist-referred patients showed the highest arthritis confirmation rate in both univariate (60.4%) and multivariable analyses (OR 2.97; 95% CI 1.20–7.36 in Model B), potentially reflecting greater proficiency in detecting synovitis on physical examination in this specialty.

Methotrexate combined with a glucocorticoid tapering regimen was the predominant first-line csDMARD recommendation, consistent with EULAR 2022 guidance [11].

### Limitations

This study has several important limitations. First, its retrospective single-centre design limits generalisability. Second, the high rate of missing serology (up to 45% for ESR) and its non-random nature introduce potential selection bias into the serological association analyses; the complete-case approach (n = 163 in Model B) may produce biased estimates if the serologically-tested subgroup differs systematically from those not tested. Third, no follow-up data are available for most patients: following the initial EAC assessment and therapy recommendation, many patients were referred back to community-based rheumatologists or GPs for ongoing management, as is common practice in the German healthcare system. Long-term outcomes including DMARD response, disease activity trajectories, and remission rates could not therefore be assessed. Fourth, both primary outcomes are clinician-dependent and subject to inter-physician variability and evolving diagnostic certainty over time. Fifth, quantitative MSUS scores were not systematically recorded in the retrospective database and therefore could not be incorporated as predictors; formal interobserver reliability of sonographic assessments was not assessed. Sixth, working diagnoses at first EAC appointment may not yet fulfil formal ACR/EULAR classification criteria, which were designed for established disease.

In conclusion, this analysis of 290 EAC patients demonstrated a 50.0% inflammatory arthritis confirmation rate via integrated clinical, serological, and sonographic assessment. Anti-CCP showed the strongest independent association with both arthritis confirmation and DMARD initiation in multivariable logistic regression. Pre-referral laboratory panels were submitted in fewer than 40% of cases, with missingness associated with referral source, representing a quality gap with direct implications for EAC efficiency. Targeted educational interventions for referring physicians and prospective data capture including quantitative MSUS scores and longitudinal outcomes are priorities for future quality improvement.

## Supplementary Information

Below is the link to the electronic supplementary material.

Supplementary file1 (DOCX 19 KB)

## Declarations

### Presentation at a congress

No part of this work has been presented at a congress or published as a congress abstract.

### AI use statement

A large language model, Claude (Anthropic; models Claude Opus 4.8), was used between May and June 2026 to assist with manuscript drafting and language editing, statistical output formatting, and reference organisation. All AI-generated content was carefully reviewed, verified against source data, and revised by the authors. All statistical analyses, clinical interpretations, and scientific conclusions are the sole responsibility of the authors. No part of this manuscript was submitted for AI generation without subsequent author review and approval.

### Disclaimer

The authors declare that no part of this report has been copied from any other publication, is currently under consideration for publication elsewhere, or has been generated by artificial intelligence without full review, verification, and revision by the authors.

### STROBE statement

This manuscript was prepared in accordance with the Strengthening the Reporting of Observational Studies in Epidemiology (STROBE) statement for cohort and cross-sectional studies. A completed STROBE checklist is submitted as a supplementary file [15].
