# Supplementary material for: Pre-referral laboratory completeness and diagnostic yield in an early arthritis clinic: a retrospective single-centre analysis
Source: Rheumatol Int. 2026 Jul 4;46(7):187. doi: 10.1007/s00296-026-06213-1 (PMC13332996; doi:10.1007/s00296-026-06213-1)
Supplement: Supplementary file 1 — Supplementary file1 (DOCX 19 KB) [file 296_2026_6213_MOESM1_ESM.docx]

**STROBE Statement—Checklist of items that should be included in reports of cohort studies**

*Manuscript: Pre-referral Laboratory Completeness and Diagnostic Yield in an Early Arthritis Clinic: A Retrospective Single-Centre Analysis. Schneider et al. 2026*

| **Item** | **Recommendation** | **Reported in (section / page)** |
| --- | --- | --- |
| **Title and abstract** | | |
| 1 | (a) Indicate the study’s design with a commonly used term in the title or the abstract. (b) Provide in the abstract an informative and balanced summary of what was done and what was found. | Title page; Abstract |
| **Introduction** | | |
| 2 | Background/rationale: Explain the scientific background and rationale for the investigation being reported. | Introduction |
| 3 | Objectives: State specific objectives, including any prespecified hypotheses. | Introduction (final paragraph) |
| **Methods** | | |
| 4 | Study design: Present key elements of study design early in the paper. | Methods – Study Design and Setting |
| 5 | Setting: Describe the setting, locations, and relevant dates, including periods of recruitment, exposure, follow-up, and data collection. | Methods – Study Design and Setting |
| 6 | (a) Eligibility criteria, sources, and methods of selection of participants; methods of follow-up. (b) For matched studies, matching criteria and number of exposed/unexposed. | Methods – Data Collection; (b) not applicable |
| 7 | Variables: Clearly define all outcomes, exposures, predictors, potential confounders, and effect modifiers. Give diagnostic criteria, if applicable. | Methods – Outcome Definitions; Data Collection |
| 8 | Data sources/measurement: For each variable of interest, give sources of data and details of methods of assessment. Describe comparability of assessment methods if more than one group. | Methods – Diagnostic Procedure; Data Collection |
| 9 | Bias: Describe any efforts to address potential sources of bias. | Methods – Statistical Analysis; Discussion – Limitations |
| 10 | Study size: Explain how the study size was arrived at. | Methods (all consecutive patients; n = 290) |
| 11 | Quantitative variables: Explain how quantitative variables were handled in the analyses; describe groupings and why. | Methods – Statistical Analysis |
| 12 | (a) Statistical methods, including those to control for confounding. (b) Methods to examine subgroups and interactions. (c) How missing data were addressed. (d) Loss to follow-up (if applicable). (e) Sensitivity analyses. | Methods – Statistical Analysis; Results – Missingness |
| **Results** | | |
| 13 | (a) Numbers of individuals at each stage. (b) Reasons for non-participation. (c) Consider a flow diagram. | Results – Patient Characteristics; Table 1 |
| 14 | (a) Characteristics of study participants and information on exposures and confounders. (b) Number with missing data per variable. (c) Summarise follow-up time. | Results; Table 1; |
| 15 | Outcome data: Report numbers of outcome events or summary measures. | Results; Table 1 (arthritis 50.0%; DMARD 44.8%) |
| 16 | (a) Unadjusted and confounder-adjusted estimates with precision (e.g., 95% CI). (b) Category boundaries when continuous variables were categorised. (c) Absolute risk for a meaningful time period if relevant. | Results – Multivariable Analysis; Tables 4 |
| 17 | Other analyses: Report other analyses done (subgroup, interactions, sensitivity). | Results – Missingness analysis |
| **Discussion** | | |
| 18 | Key results: Summarise key results with reference to study objectives. | Discussion (opening); Key Messages |
| 19 | Limitations: Discuss limitations, taking into account sources of potential bias or imprecision; direction and magnitude of any potential bias. | Discussion – Limitations |
| 20 | Interpretation: Give a cautious overall interpretation considering objectives, limitations, multiplicity of analyses, results from similar studies, and other relevant evidence. | Discussion (final paragraphs) |
| 21 | Generalisability: Discuss the generalisability (external validity) of the study results. | Discussion – Limitations |
| **Other information** | | |
| 22 | Funding: Give the source of funding and the role of the funders for the present study and, if applicable, for the original study on which the present article is based. | Declarations – Funding |
